# Chemokines CCL2, 3, 14 stimulate macrophage bone marrow homing, proliferation, and polarization in multiple myeloma

**DOI:** 10.18632/oncotarget.4523

**Published:** 2015-06-19

**Authors:** Yi Li, Yuhuan Zheng, Tianshu Li, Qiang Wang, Jianfei Qian, Yong Lu, Mingjun Zhang, Enguang Bi, Maojie Yang, Frederic Reu, Qing Yi, Zhen Cai

**Affiliations:** ^1^ Bone Marrow Transplantation Center, Department of Hematology, Zhejiang University, Hangzhou, Zhejiang, China; ^2^ Department of Cancer Biology, Lerner Research Institute, Cleveland Clinic, Cleveland, OH, USA; ^3^ Department of Hematology, Sichuan University, West China School of Medicine, Chengdu, Sichuan, China; ^4^ Taussig Cancer Institute, Cleveland Clinic, Cleveland, OH, USA

**Keywords:** multiple myeloma, chemokine, macrophage, bone marrow

## Abstract

We previously showed that macrophages (MΦs) infiltrate the bone marrow (BM) of patients with myeloma and may play a role in drug resistance. This study analyzed chemokines expressed by myeloma BM that are responsible for recruiting monocytes to the tumor bed. We found that chemokines CCL3, CCL14, and CCL2 were highly expressed by myeloma and BM cells, and the levels of CCL14 and CCL3 in myeloma BM positively correlated with the percentage of BM-infiltrating MΦs. *In vitro,* these chemokines were responsible for chemoattracting human monocytes to tumor sites and *in vivo* for MΦ infiltration into myeloma-bearing BM in the 5TGM1 mouse model. Surprisingly, we also found that these chemokines stimulated MΦ *in vitro* proliferation induced by myeloma cells and *in vivo* in a human myeloma xenograft SCID mouse model. The chemokines also activated normal MΦ polarization and differentiation into myeloma-associated MΦs. Western blot analysis revealed that these chemokines promoted growth and survival signaling in MΦs via activating the PI3K/Akt and ERK MAPK pathways and c-myc expression. Thus, this study provides novel insight into the mechanism of MΦ infiltration of BM and also potential targets for improving the efficacy of chemotherapy in myeloma.

## INTRODUCTION

Multiple myeloma (MM) is characterized by accumulation of monoclonal malignant plasma cells in the bone marrow (BM). In the United States, about 22,000 patients are newly diagnosed yearly, and MM accounts for 20% of deaths in all hematological malignancies [1]. MM management has improved in the past decades, mostly due to the use of new drugs in clinic. However, MM remains an incurable disease, and the current survival is approximately 6-8 years [2]. One major problem in MM management is that MM cells develop drug resistance within the BM microenvironment [2]. Our previous research has suggested that macrophages (MΦs) are an important component in the MM-BM microenvironment. MΦ infiltration is increased in MM BM, and these MM-associated MΦs (mMΦs) induce MM drug resistance *in vitro* and *in vivo* in MM mouse models [3, 4]. Suyani et al have shown that MM patients with high BM MΦ infiltration have poor prognosis [5]. All these findings suggest that mMΦ may be a risk factor in MM management.

Findings from tumor-associated MΦs (TAMs) in human solid cancers suggest that most TAMs originate from circulating monocytes (MOs) [6, 7]. Cells in the tumor microenvironment, including both tumor cells and stromal cells, overexpressed chemokines such as CCL2 (MCP-1), CXCL12 (SDF-1), CCL9 (MIP-1γ) and/or CCL18 (PARC), and recruit MOs into the tumor bed. Recruited MOs differentiate into TAMs in the presence of MΦ differentiation factors such as CSF-1, GM-CSF, and Flt3-ligand [6, 8]. Interestingly, the MΦ differentiation factor CSF-1 may also regulate MO chemotaxis to the tumor bed, suggesting crosstalk between MO recruitment and TAM differentiation [9]. In addition to MO chemotaxis into the tumor bed, resident TAM division also contributes to the increased numbers of TAMs in tumor sites [10]. However, the mechanisms underlying the increased numbers of MΦs and polarization of normal MΦs to mMΦs in MM BM are unclear. In this study we analyzed the chemokines expressed in the MM BM microenvironment and their roles in recruiting MOs to the MM tumor bed and conditioning them to become mMΦs.

## RESULTS

### Human myeloma bone marrow overexpresses chemokines CCL2, CCL3, and CCL14

**Figure 1:**
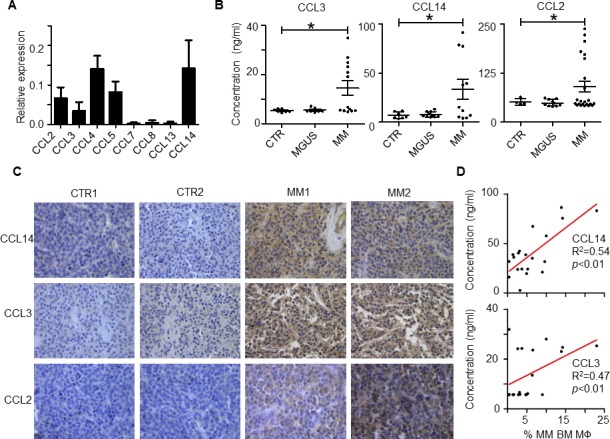
Expression of MO chemokines in human MM BM **A.** qPCR analysis of expression of different MO chemokines in MM patient BM cells. The value indicates the relative expression to GAPDH. One representative sample of 4 patient samples analyzed is shown. **B.** Levels of CCL3, CCL14, and CCL2, measured by ELISA, in BM plasma of healthy donors (CTR) and patients with MM or MGUS. The numbers of CTR, MGUS and MM patients used for measuring CCL2 are 4, 10, and 23, respectively; for CCL14 are 7, 10, and 11, respectively; and for CCL3 are 7, 10, and 13, respectively. **C.** Immunohistochemistry analysis of CCL3, CCL14, and CCL2 expression in BM biopsies of 2 healthy donors (CTR1 and CTR2) and 2 representatives (MM1 and MM2) out of five MM patients. **D.** Linear regression analysis of the relationship between the percentage of BM MΦs and concentration of chemokines CCL14 (*n* = 20) and CCL3 (*n* = 18) in BM plasma in MM patients. *p < 0.05.

To identify chemokines that regulate MO/MΦ chemoattraction to the MM tumor bed, we examined expression of different MO chemokines in MM BM cells (total cells from MM BM aspirates) by qPCR [11]. As shown in Figure 1A, expression of CCL2, 3, 4, 5, 7, 8, 13, and 14 varied in MM BM. Among them, CCL2, 3, 4, 5, and 14 had relatively high expression. Next, we hypothesized that only the chemokines that were overexpressed in MM BM, but not in healthy BM, might contribute to the increased MΦ accumulation in MM tumor bed. Thus, we compared the chemokine expression profiles in MM BM vs. healthy BM plasma by ELISA. CCL3 (MIP-1α), CCL14 (HCC1), and CCL2 (MCP-1) were highly expressed in BM plasma from MM patients, but not in BM from healthy donors (Figure 1B; *p* < 0.05). The expression of CCL5 (RANTES) or CCL4 (MIP-1β) was no different between the patient and healthy donor samples (*p* > 0.05). Immunohistochemistry analysis of human BM biopsies also confirmed that MM BM highly expressed CCL3, CCL14, and CCL2 proteins (Figure 1C).

Finally, we analyzed the association between chemokine expression in BM plasma and the number of BM MΦs in MM patients. BM plasma chemokine expression was determined by ELISA, and the number of MΦs was measured by flow cytometry for CD14^+^/CD68^+^ cells as previously described [4]. As shown in Figure 1D, linear regression revealed that MM patients with high CCL14 and CCL3 levels in BM also had a high percentage of BM MΦs (*p* < 0.01). No positive correlation was found between CCL2 expression and the percentage of BM MΦs (*p* > 0.05). Overall, our results suggested that chemokines CCL3, CCL14, and CCL2 were highly expressed in MM BM compared with normal BM, and CCL3 and CCL14 expression levels positively correlated with the numbers of BM MΦs in MM patients.

### Human myeloma cells upregulate the expression of chemokines by bone marrow stromal cells

**Figure 2:**
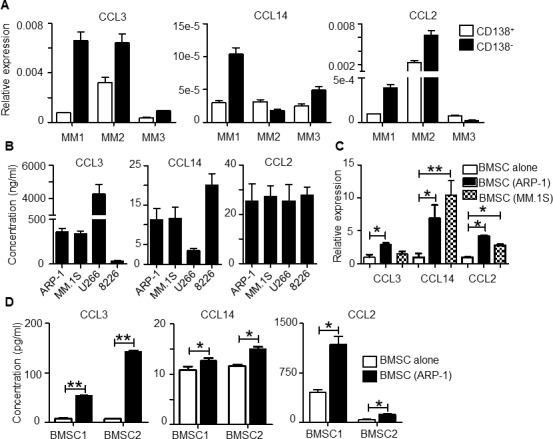
Expression of CCL2, CCL3 and CCL14 by human MM BM cells **A.** qPCR analysis of CCL3, CCL14, and CCL2 expression in CD138^+^ primary MM cells and CD138^−^ non-malignant cells in BM aspirates from 3 different MM patients (MM1 to MM3). The y-axis indicates the fold change relative to GAPDH value. **B.** Concentration of chemokines secreted by 4 human MM cell lines. MM cells (2 ×10^5^ cell per well) were cultured *in vitro* for 24 hours; then the chemokine expression in culture supernatant was analyzed by ELISA. **C.** BMSCs were cocultured with ARP-1 or MM.1S MM cells for 48 hours. CCL3, CCL14, and CCL2 expression in BMSCs was analyzed by qPCR. **D.** BMSCs were cultured in transwells with MM cells ARP-1 for 48 hours, followed by removal of MM cells and cultured alone in fresh medium for 24 hours. BMSCs from the same donors were cultured alone in parallel as controls. Chemokine concentrations were determined by ELISA using culture supernatants collected at the end of culture. *p < 0.05, **p < 0.01.

As the MM BM microenvironment consists of both (CD138^+^) malignant plasma cells and (CD138^−^) non-malignant cells, we examined the sources of cells that produce the identified chemokines CCL3, CCL14 and CCL2. RT-PCR analysis of MM patient samples showed that both CD138^+^ and CD138^−^ cells in MM BM expressed CCL3, CCL14, and CCL2 and that, for the most part, expression was higher in CD138^−^ cells than CD138^+^ primary MM cells (Figure 2A). MM cell lines also expressed (data not shown) and secreted these chemokines (Figure 2B). On the other hand, in MM cell (ARP-1 and MM.1S)–conditioned bone marrow stromal cells (BMSCs), expression of these chemokines was upregulated at both mRNA and protein levels compared with unconditioned BMSCs (Figure 2C and 2D, *p* < 0.05 to *p* < 0.01). Overall, our results suggested that both MM cells and BMSCs, especially MM-associated BMSCs, contributed to secretion of high levels of CCL3, CCL14 and CCL2 chemokines in the MM BM microenvironment.

### CCL3, CCL14 and CCL2 activate monocyte migration to the MM tumor bed *in vitro*

**Figure 3:**
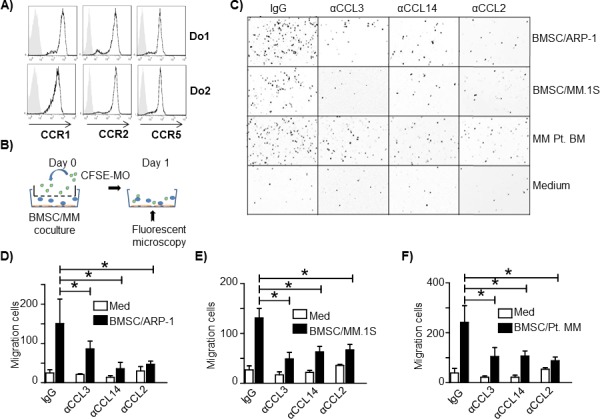
CCL2, CCL3 and CCL14 activate human MO migration to MM tumor bed **A.** Flow cytometry analysis of chemokine receptors CCR1, CCR2 and CCR5 expression on MOs from healthy donors. Two representatives of three samples analyzed are shown. **B.** Schematic diagram depicting the *in vitro* migration assay. **C.** Representative image of MOs/MΦs that migrated through the transwell to the lower chambers containing cocultured BMSCs and the MM cell lines ARP-1 or MM.1S, MM BM aspirate cells from a patient (MM Pt. BM), or medium alone. The image is color reversed to emphasize migrated cells (dark dots) in lower chamber (white background). One representative image out of three performed experiments is shown. Quantification of *in vitro* MO migration toward **D.** BMSC/ARP-1 cells, **E.** BMSC/MM.1S cells, or **F.** primary BM cells from three MM patients. *p < 0.05.

Flow cytometry analysis showed that MOs expressed CCL3 receptors CCR1 and CCR5; CCL2 receptor CCR2, and CCL14 receptors CCR1 and CCR5 (Figure 3A). Next we examined the role of CCL3, CCL14, and CCL2 in MO chemoattraction using an *in vitro* cell migration assay. As shown in Figure 3B, MM cell lines (ARP-1 and MM.1S) were cocultured with human BMSC or total primary MM BM cells were cultured alone in the lower chamber to simulate the MM tumor bed. MOs isolated from healthy donor peripheral blood mononuclear cells were labeled with CFSE green fluorescent dye and added in the upper transwell chamber. After coculture for 24 hours, MOs migrating to the lower chamber were identified by fluorescence microscopy and quantified. MM and BMSC coculture and total BM cells from MM patients stimulated greater MO migration than medium control (Figure 3C), indicating that MM BMSC together could chemoattract MOs to tumor sites. Addition of CCL3, CCL14, or CCL2 neutralizing antibodies (a final concentration of 10 μg/ml for each antibody) inhibited MO migration (Figure 3D-3F, *p* < 0.05). Thus, our data indicated that MOs were attracted to the MM tumor bed by chemokines CCL3, CCL14 or CCL2 because the process was significantly attenuated by antibodies directed against these chemokines.

### CCL3 and CCL2 promote bone marrow macrophage infiltration *in vivo* in murine myeloma mouse model

**Figure 4:**
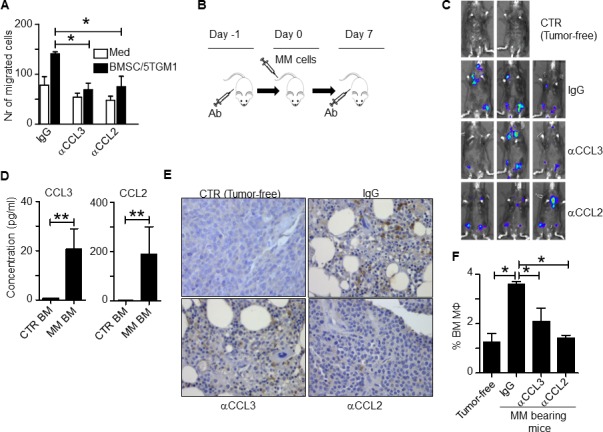
CCL2 and CCL3 promote murine MO infiltration in vivo **A.** *In vitro* migration assay of mouse BMSC and 5TGM1 cells. Medium alone served as control. Control IgG or neutralizing antibodies to chemokines CCL3 or CCL2 were added to the cultures. **B.** Schematic diagram depicting the schedule of animal study. **C.** *In vivo* bioluminescent imaging showing locations of tumor formation in mice treated with control IgG or various chemokine neutralizing antibodies. CTR indicates tumor-free mice. **D.** Concentrations of chemokines secreted by BM cells from rear leg bones of tumor-free mice (CTR BM) and tumor-bearing mice (MM-BM). BM cells were harvested and cultured *ex vivo* for 24 hours. The chemokine expression in culture supernatant was analyzed by ELISA; **E.** Immunohistochemistry staining for F4/80, a mouse MΦ marker, in BM sections from tumor-free mice (CTR) or MM-bearing mice treated with control IgG or neutralizing antibodies against CCL3 or CCL2. Compared to BM from tumor-free control mice, MM-BM had greater MΦ infiltration (brown; MΦ staining was intermediate in both CCL3 and CCL2 neutralizing antibody-treated tumor-bearing mice). **F.** Flow cytometry analysis showing the percentages of CD14^+^ and F4/80^+^ double positive MΦs in BM cells from tumor-free mice or MM-bearing mice treated with control IgG or neutralizing antibodies against CCL3 or CCL2. *p < 0.05, **p < 0.01.

To study the role of the identified chemokines in MO and MΦ recruitment *in vivo*, we used the murine 5TGM1 MM model established in C57BL/KawRij mice [12]. Because the mouse does not have CCL14 homology and human CCL14 cDNA shares the highest similarity with human and mouse CCL3 (data not shown), we first examined *in vitro* murine MΦ chemotaxis in the presence of neutralizing antibodies to murine CCL3 or CCL2. 5TGM1 cells were cocultured with mouse BMSCs, generated from C57BL/KawRij mouse hind leg BM, in the lower transwell chamber, and mouse peritoneal MΦs were added in the upper chamber. As shown in Figure 4A, CCL3 or CCL2 neutralizing antibodies attenuated murine MΦ chemotactic migration *in vitro* (*p* < 0.05), confirming the role of these murine chemokines in MΦ chemotactic migration. Next, we intravenously inoculated 5TGM1 cells into the mice and treated them with either control IgG or neutralizing antibodies against CCL3 or CCL2 (100 μg per mouse each time, intraperitoneally injected 1 day before and 7 days after tumor cell inoculation; Figure 4B). Tumor development was monitored every 7 days by *in vivo* bioluminescent assay and by measuring circulating mouse IgG2b that was secreted by 5TGM1 cells (data not shown). Mice treated with control IgG or neutralizing antibodies showed similar tumor burdens (Figure 4C), probably because MOs/MΦs promote MM chemoresistance without affecting tumor growth, particularly at an early stage of disease. However, when we compared the expression of the chemokine genes in MM BM (5TGM1 tumor-bearing mice) versus tumor-free BM, we found that CCL3 and CCL2 expression was higher in MM BM (Figure 4D, *p* < 0.01).

Immunohistochemistry staining for F4/80, a murine MΦ marker, in mouse BM showed that MM BM had a greater percentage of BM MΦs, similar to what we observed in human MM BM [3], and injection of CCL3 or CCL2 neutralizing antibodies significantly reduced the percentage of MΦs in murine MM BM (Figure 4E). This finding was confirmed by flow cytometry analysis of mouse BM cells to quantify the numbers of CD14^+^ and F4/80^+^ MΦs (Figure 4F, *p* < 0.05). Taken together, these *in vivo* findings strongly suggested that chemokines CCL3 and CCL2 were responsible for attracting MOs to infiltrate MM BM microenvironment *in vivo*.

### Human myeloma cells enhance monocyte/macrophage proliferation *in vitro* and *in vivo*

**Figure 5:**
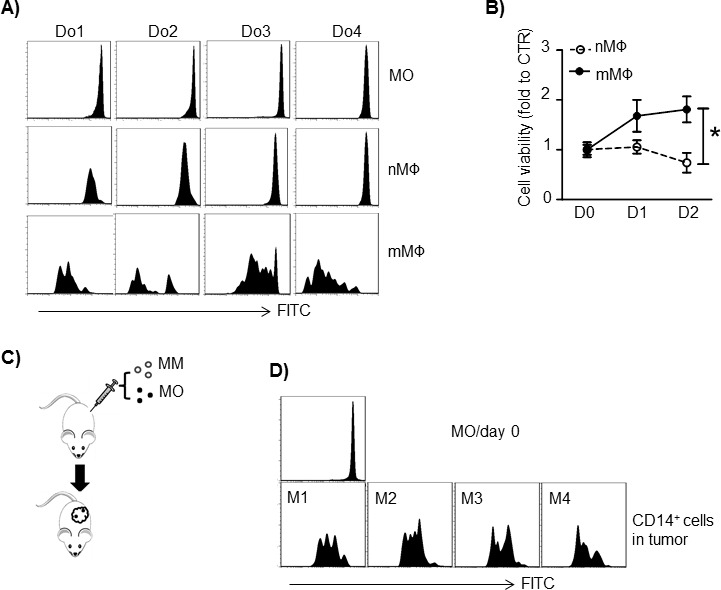
Human MM cells stimulate MΦ proliferation **A.** MOs from 4 healthy donors (Do1 to Do4) were labeled with CFSE and cultured *in vitro* to generate nMΦs. mMΦs were generated by transwell coculture nMΦs with MM cells ARP-1. The CFSE intensity in CD14^+^ cells was examined by flow cytometry. **B.** MΦ proliferation measured by MTS assay. *In vitro*-generated human normal MΦ (nMΦ) and MM-associated MΦ (mMΦ) were cultured in medium alone for 2 days, and cell proliferation was measured daily. The control value was set to 1.0. **C.** Schematic diagram depicting human MM-xenografted SCID model injected with human MOs. MOs were labeled with CFSE before subcutaneous injection. **D.** CD14^+^ cell CFSE intensity in subcutaneous tumors harvested on day 21 after tumor injection. Results from 4 mice injected with MOs from 4 healthy donors (Do1 to Do4) are shown. *p < 0.05.

Previous studies showed that MΦ *in situ* proliferation also contributes to increased numbers of tissue MΦs and TAMs [10]. Therefore, we investigated MO and MΦ proliferation in the MM tumor bed. First, we examined MM-conditioned mMΦ proliferation *in vitro*. MOs were prestained with the fluorescent dye CFSE to identify nMΦs. nMΦs were further transwell-cocultured with MM cells (ARP-1 cells) to obtain mMΦs. Diluted CFSE signals were observed in mMΦs but not in nMΦs cultured alone (Figure 5A), indicating that MM cells stimulated nMΦs to proliferate. In a different experiment, established nMΦ and mMΦ were cultured in medium for 2 days. Cell proliferation was assessed by MTS assay. mMΦs but not nMΦs proliferated in 2-day culture (Figure 5B; *p* < 0.05). Finally, we examined whether human MOs/MΦs could proliferate *in vivo* in a human MM xenograft mouse model created as we have described previously [4]. MM tumor cells (ARP-1) were subcutaneously inoculated into SCID mice together with CFSE-labeled MOs obtained from healthy donors (Figure 5C). After tumor development, the human MOs polarized into mMΦs. Cells in the tumor bed were assessed by flow cytometry analysis for CD14^+^/CFSE^+^ expression. As shown in Figure 5D, human mMΦs in the tumor bed exhibited a diluted CFSE signal, indicating cell division occurring *in vivo*. Overall, our results suggested that MM cells enhanced MΦ proliferation *in vitro* and *in vivo*.

### Human CCL3, CCL14 and CCL2 stimulate monocyte/macrophage proliferation and differentiation

**Figure 6:**
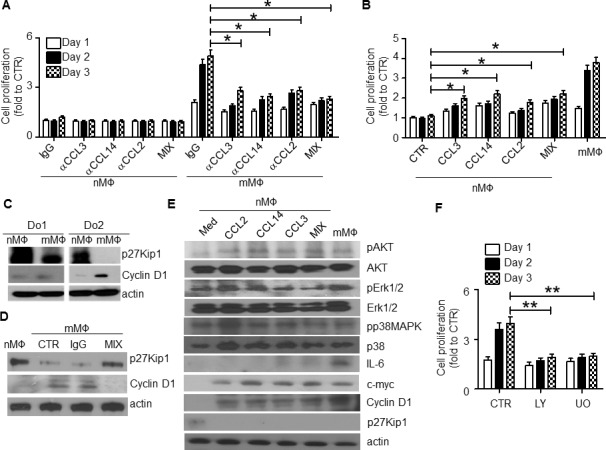
CCL2, CCL3 and CCL14 stimulate human MΦ proliferation and intracellular signaling **A.** nMΦ or mMΦ were generated as described in the Methods. The cells were then cultured *in vitro* in the presence of each of the chemokine neutralizing antibodies (αCCL3, αCCL14 and αCCL2, at a final concentration of 10 μg/ml each), and their combination (MIX). An equal amount of IgG was used as control. Cell proliferation at different time points was examined by MTS. **B.** nMΦs were cultured for 3 days in the presence of each of the recombinant chemokines (final concentration of 1 μg/ml) or their combination (MIX). mMΦs cultured in medium alone were used as a positive control. Cell proliferation at different time points was determined by MTS assay. **C.** Western blot showing the levels of p27Kip1 and cyclin D1 in nMΦs and mMΦs from 2 healthy donors (Do1 and Do2). **D.** Western blot showing the levels of p27Kip1 and cyclin D1 in nMΦs and mMΦs. mMΦs were generated in coculture with MM cells in transwells in the absence (CTR) or presence of control IgG or a combination of the chemokine neutralizing antibodies (MIX). **E.** Western blot showing the levels of different kinases, IL-6, c-myc, cyclin D1, and p27Kip1 in nMΦs in overnight cultures in medium or with addition of each of the chemokines CCL2, CCL14, or CCL3 individually or all three combined (MIX). mMΦs were used as a positive control. **F.** Proliferative response of mMΦs in medium alone (CTR) or in the presence of the PI3K-Akt inhibitor LY294002 (LY; 50 μM) or Erk1/2 inhibitor U0126 (UO; 2 μM). *p < 0.05, **p < 0.01.

Next we examined the crosstalk between the chemokine-mediated MO/MΦ chemoattraction and mMΦ proliferation. Surprisingly, our results suggested that the chemokines CCL3, CCL14, and CCL2 were also involved in regulating mMΦ proliferation. MΦs were obtained from healthy blood donors and used as nMΦs, or cocultured in transwells with MM cells for 3 days to generate mMΦs. As shown in Figure 6A, mMΦs but not nMΦs proliferated, which could be inhibited by CCL3, CCL14, or CCL2 neutralizing antibodies, added alone or in a combination at the time of coculture (*p* < 0.05). On the other hand, addition of CCL3, CCL14, or CCL2 could also stimulate nMΦs to proliferate (Figure 6B; *p* < 0.05). Western blot analysis suggested that the increased mMΦ proliferation might be caused by upregulated expression of cyclin D1 and downregulated p27Kip1 (Figure 6C), which are positive and negative, respectively, regulators of cell cycle progression. The chemokine neutralizing antibodies altered cyclin D1 and p27Kip1 expression in mMΦs (Figure 6D), suggesting that these chemokines were responsible for upregulated cyclin D1 and downregulated p27Kip1. Furthermore, mMΦs displayed activated PI3K-Akt and MAPK/Erk pathways and upregulated c-myc and IL-6 expression (Figure 6E). Similarly, addition of these chemokines to nMΦ could activate these signaling pathways. As upregulated c-myc and IL-6 expression are considered hallmarks for TAMs or mMΦs [3, 13], these findings suggested that these chemokines could also polarize nMΦs to mMΦs. Finally we showed that the PI3K-Akt inhibitor LY294002 and MAPK/Erk inhibitor U0126 reduced mMΦ proliferation (Figure 6F; *p* < 0.01). Taken together, our findings suggested that the chemokines CCL2, CCL3 and CCL14 not only regulated MO/MΦ chemoattraction, but also controlled mMΦ proliferation within the MM tumor bed and induced MΦ polarization toward mMΦs.

## DISCUSSION

MΦs are major tumor-infiltrating myeloid cells. As an abnormally differentiated cell type induced by both intrinsic and extrinsic stimuli, TAMs have tumor-promoting phenotype as they activate tumor angiogenesis, cancer cell proliferation, immune-suppression [14], cancel cell survival, and metastasis [6]. The abundance of TAMs usually indicates poor prognosis in various human cancers [6]. The origin of TAMs has been widely investigated in different human cancers. Although the prevailing hypothesis regarding tissue MΦ origination has been questioned recently, it is still widely accepted that TAMs originate from circulating MOs via chemoattraction into the tumor bed by chemokines that are secreted by tumor cells and/or tumor stroma [10, 15]. To investigate which chemokines regulate MO/MΦ infiltration in MM BM, we examined expression of different chemokines in MM BM aspirates. Three chemokines that attract MOs and MΦs, CCL3, CCL14, and CCL2, were overexpressed in MM BM as compared with normal BM, most likely secreted by both MM cells and MM BMSCs. Importantly, we found that CCL3 and CCL14 levels in MM patient BM plasma positively correlated with the number of patient BM MΦs. *In vitro* and *in vivo* studies suggested that CCL3, CCL14 and CCL2 could promote MO chemotactic migration into MM BM.

Among those identified chemokines, CCL2 has long been recognized as a regulator of TAMs in different human cancers [16], such as breast cancer [17] and colon cancer [18]. In general, tumor cells and stroma overexpress CCL2, which results in increased MO recruitment and TAM generation. Also, Sinya and colleagues showed that CCL2 deficiency decreased adipose tissue MΦ proliferation *in vivo,* and the *in situ* proliferation of resident MΦs driven by CCL2 is a crucial process for MΦ accumulation in adipose tissue in obesity [19]. Furthermore, Elena and colleagues showed that blocking CCL2 led to M1 polarization-associated gene and cytokine upregulation and diminished expression of M2-associated markers in human MΦs. In CCL2 receptor CCR2-deficient mice, BM-derived MΦs were reported to display an M1-like polarization profile at the transcription level. This CCL2-CCR2 axis shaped MΦ polarization [20]. The C-myc oncogene is a key player in alternative MΦ activation [21]. Interestingly, we found that CCL2 induced c-myc expression in MΦs. CCL3 has also been reported for MO/MΦ chemoattaction in hepatocarcinoma [22] and pancreatic carcinoma [23]. Different chemokines may play redundant roles within the same tumor setting. For example, both the MO chemokines CCL2 and CCL5 are overexpressed in breast cancer [24]. In MM, CCL3 and CCL2 have been found to regulate osteoclast-mediated bone resorption. CCL3 and CCL2 bind to their receptors expressed on osteoclast precursors and promote osteoclast formation [25, 26]. CCL3 and CCL2 overexpression in MM has also been reported previously [27, 28]. Unlike CCL3 or CCL2, CCL14 function in MM has not been reported. CCL14 is unique in primates and has no corresponding homology in mouse or rat [29]. CCL14 chemotactic activity on MOs is relatively low until this chemokine is proteolytically processed by serine proteases, after which the CCL14 chemotactic activity on MOs increases 100-1000 fold [30]. Very few studies have been published that address CCL14 function in human cancer. Michiels et al. showed that CCL14 was increased in the oral fluids of head and neck cancer patients [31]. Li et al suggested that CCL14 might promote angiogenesis and metastasis of breast cancer [32]. In this study, we found that CCL14 was overexpressed in MM BM and regulated MO recruitment to the tumor bed.

CCL3, CCL14 and CCL2 all belong to the CC chemokine family. CCL3 and CCL14 share the same receptors, CCR1 and CCR5, whereas CCL2 binds to CCR2 [11]. All these CCRs are G protein-coupled transmembrane receptors that can initiate intracellular signaling transduction depending on the cell type and ligand-receptor axis specificity [33]. In our study, we showed that in MΦs, CCL3, CCL14, and CCL2 activated the PI3K-Akt and MAPK/Erk pathways, both of which promote cell proliferation. Previous studies have shown that downstream of activation CCR1, the receptor for CCL3, CCL14 and many other chemokines, leads to PI3K-Akt [34] and MAPK/Erk signaling [35]. In addition, CCR2 may also initiate intracellular MAPK/Erk activation [36]. Although there is no evidence for CCR5-induced PI3K-Akt or MAPK/Erk activation, CCR5 may alter cell signaling by activating the JNK and p38MAPK pathways [37]. Both PI3K-Akt and MAPK/Erk pathways are closely related to cell proliferation. Zhou et al has shown that activation of Akt results in upregulation of cyclin D and downregulation of p27 in BM MΦs [38]. Therefore, upon binding to their receptors, CCL3, CCL14, and CCL2 not only lead to MO/MΦ chemoattraction, but may also promote MO/MΦ proliferation and polarization to mMΦs.

To summarize, we analyzed MO/MΦ chemokines expressed in MM BM, and identified CCL3, CCL14, and CCL2 as functional chemokines that were responsible for increased MΦ infiltration in the MM BM microenvironment. We also found that these chemokines promoted mMΦ proliferation within the tumor bed and polarization of normal MΦs to mMΦs via activation of the PI3K-Akt and MAPK/ERK pathways. Because mMΦs confer MM chemoresistance, targeting those chemokines may prevent MΦ infiltration in the tumor bed, and therefore may improve the efficacy of MM chemotherapy.

## MATERIALS AND METHODS

### Cells

Human MM cell lines MM.1S, ARP-1, RPMI-8226 and U266 were cultured in RPMI-1640 medium with 10% fetal bovine serum (FBS), 100 U/ml penicillin, and 100 μg/ml streptomycin at 37°C and 5% CO_2_. Mouse 5TGM1 myeloma cells expressing the luciferase gene were kindly provided by Dr. Frederic Reu, Cleveland Clinic. 5TGM1 cells were cultured in IMDM medium with 10% FBS and antibiotics. Primary MM cells from MM patients were purified by CD138^+^ magnetic beads (Stemcell Technologies).

Human (patients and healthy volunteers) MΦs were generated from the buffy coat as described previously [3]. In general, buffy coat mononuclear cells were incubated in 6-well plates for 1-2 hours at 37° to remove nonadherent cells. Adherent cells were incubated for 5-7 days in media containing recombinant human M-CSF (10 ng/ml) to promote conversion to normal macrophages (nMΦs). Polarization to mMΦs was achieved by coculturing nMΦs in a transwell system with ARP-1 or MM.1S MM cells for another 48-72 hours.

In some experiments, MOs were isolated from buffy coat using MO enrichment kit (Stemcell). For MO proliferation assay, purified MOs were labeled with CFSE, seeded in 24-well plates, and cocultured with or without MM cells for 7 days. For MO migration assay, CFSE-labeled MOs were directly used in the assay.

Human BMSCs were obtained from BM aspirates and cultured *in vitro* as previously described [39]. In brief, BM mononuclear cells from healthy donors were seeded and cultured in RPMI-1640 medium with 10% fetal bovine serum, 100 U/ml penicillin, and 100 μg/ml streptomycin. Medium was changed every 2-4 days, and adherent cells were maintained in culture. Early passage cells (< 5 passages) were used in the experiment.

### Patient samples and controls

BM aspirates were from of newly diagnosed patients with MM or MGUS. BM aspirates from healthy donors were used as controls. The same patients and volunteers also provided blood samples to generate mononuclear cells. All of the human participants provided written informed consent, and this study was approved by the Institutional Review Board at Cleveland Clinic and the Ethics Committees of the First Affiliated Hospital of Zhejiang University, Medical College. The plasma of MM BM aspirates was used for ELISA detection of chemokines. Cells from BM aspirates were used for flow cytometry analysis of MΦs. CD68 and CD14 double positive cells were considered as BM MΦs.

### Animals

The 5TGM1 MM mouse model was used for the animal study. 5TGM1 cells were intravenously inoculated into 6-week-old C57BL/KawRij mice (1 million cells/mouse), and tumor development was monitored by luciferase activity using an *in vivo* imaging system (IVIS, PerkinElmer). Mouse plasma was collected at specified time points when animals were euthanized, and tumor burden was also monitored by measuring mouse IgG2b by ELISA. The percentage of CD138^+^ MM cells in rear leg BM was determined by flow cytometry. In some experiment, peritoneal MΦs were isolated from C57BL/KawRij mice. In brief, mice were euthanized and a small incision was made on the abdomen skin. Sterile PBS was injected into the caudal to suspend residual cells. The cell suspension was harvested and spun down for peritoneal MΦs. To determine proliferation of human MΦs in the tumor bed *in vivo*, a human MM xenograft SCID mouse model was established as previously described [4] by injecting human MOs together with MM cells. All experiments complied with protocols approved by IACUC committee at the Cleveland Clinic.

### Antibodies and reagents

Neutralizing antibodies to human CCL3 (MAB270), CCL14 (MAB3241) and CCL2 (MAB679), and mouse CCL3 (AB-450-NA), CCL2 (AB-479-NA) were purchased from R&D Systems. Immunohistochemistry antibodies to human CCL3, CCL14 and CCL2 and mouse F4/80 were purchased from Santa Cruz Bio. Western blot antibodies against cyclin D1, p27Kip1, c-myc, pAkt, Akt, pErk, Erk, IL6, c-myc, pp38MAPK, p38MAPK, and α-actin were from Cell Signaling. FITC-, PE, and APC-conjugated monoclonal antibodies to human CD14, CD68, CCR1, CCR2, CCR5 and mouse CD14, CD138, F4/80 were from purchased Biolegend.

The CellTrace CFSE kit was purchased from Invitrogen. Cell proliferation was analyzed using an MTS kit (Promega) following the standard protocol. Propidium iodide (PI) and RNase were purchased from Sigma Aldrich.

### Enzyme-linked immunosorbent assay (ELISA)

Chemokines in BM aspirates or cell culture supernatant (kits from R&D Systems) and circulating mouse IgG2b in mouse plasma (kits from eBioscience) were analyzed by ELISA.

### Chemotactic assay

When BMSCs reached 70% confluence, they were directly cocultured with MM cells (2 × 10^5^ cells per well) in 24-well plates. CFSE-labeled MOs were put in transwell inserts (pore size: 5 μm, Corning) and cocultured with MM/BMSC or primary MM BM cells overnight. MOs migrating to the lower chamber were detected by fluorescent microscopy (50 ×, Leica) for CFSE^+^ cells. MOs migration to the lower chamber in medium only was the control. For quantification of the results, migrated cells in three randomly chosen view zones were counted and the mean value was used for plotting. In some experiments, chemokine neutralizing antibody or control IgG were added in the coculture (a final concentration of 10 μg/ml) to neutralize the chemokines.

### Immunohistochemistry staining

Paraffin-embedded human MM and healthy BM biopsies (US Biomax, Rockville, MD, USA) and mouse tissues were used for immunohistochemistry staining with a kit (Vectastain ABC Kit, Vector Laboratories) following manufacturer's instructions. For murine myeloma tissue preparation, the mouse hind leg bone was fixed in 4% paraformaldehyde solution for 3 days. After fixation, the bone was socked in decalcification solution (10% EDTA solution) for 3-4 weeks before paraffin embedding and section.

### Real-time PCR

Total RNA was extracted from cells with RNeasy Mini Kit (QIAGEN) according to the manufacturer's instruction. The expressions of target genes were analyzed by qPCR using SYBR green real-time PCR system (StepOne Plus, Applied Bio systems). The expression of housekeeping gene GAPDH was used as an internal control. Primer used as follows (primer sequence 5′-3′): Forward: ACATCTCACAAAGCATCCCG and Reverse: TCATGCAATCCTGAACTCCC for human CCL14; Forward: TGTCCCAAAGAAGCTGTGATC and Reverse: ATTCTTGGGTTGTGGAGTGAG for human CCL2; Forward: AACCTTCACCTCTCATGCTG and Reverse: TGGAAACTGAATCTGGCTGAG for human CCL8; Forward: AGACCAAACCAGAAACCTCC and Reverse: AGTATTAATCCCAACTGGCTGA for human CCL7; Forward: CACTCAACGTCCCATCTACTTG and Reverse: AGATCTCCTTGCCCAGTTTG for human CCL13; Forward: CGGCAGATTCCACAGAATTTC and Reverse: AGGTCGCTGACATATTTCTGG for human CCL3; Forward: TCCTCGCAACTTTGTGGTAG and Reverse: TTCAGTTCCAGGTCATACACG for human CCL4; Forward: CTGCTTTGCCTACATTGCCC and Reverse: CTTGTTCAGCCGGGAGTCAT for human CCL5; Forward: ACAGTCAGCCGCATCTTC and Reverse: CCACTTTACCAGAGTTAAAAGCAG for human GAPDH.

### Statistical analysis

Two-tailed Student's t-test was used for statistical analysis. A *p* value less than 0.05 was considered statistically significant. Results are presented as means ± SD of at least three different experiments, unless otherwise indicated.
